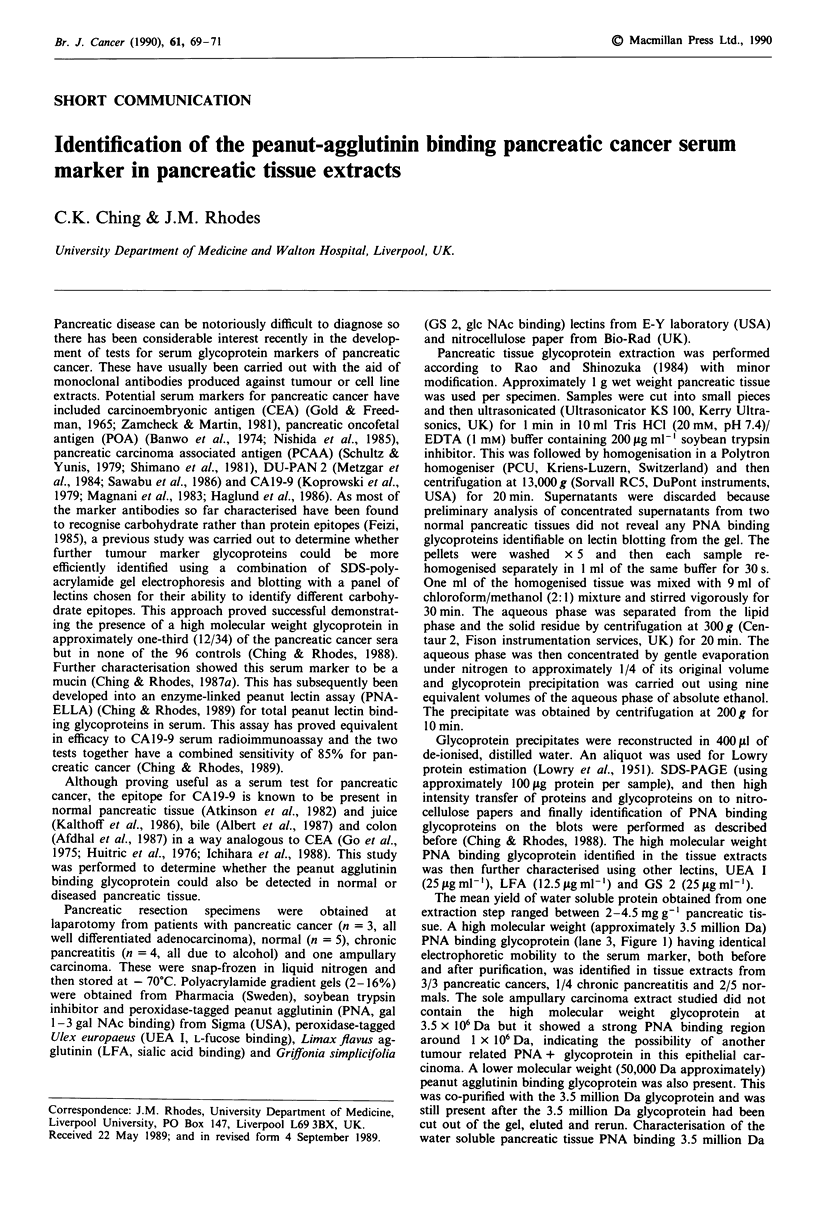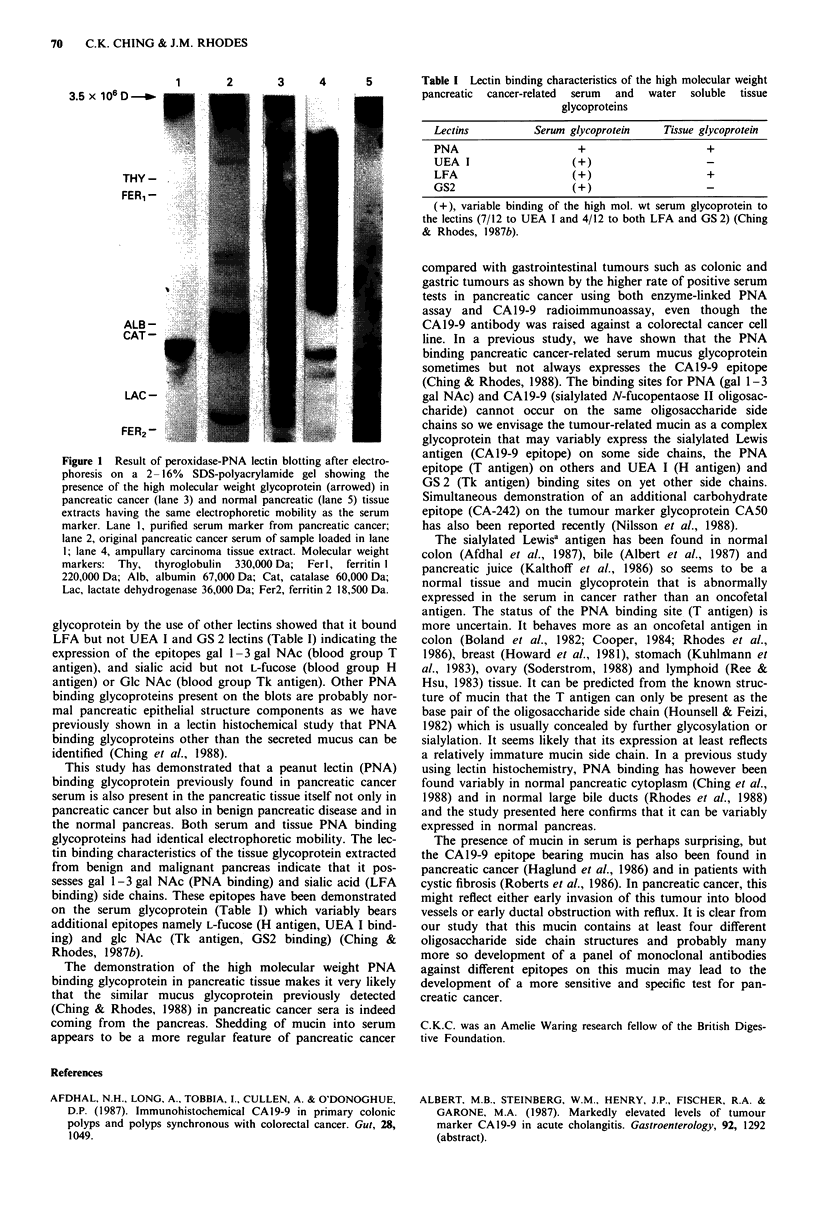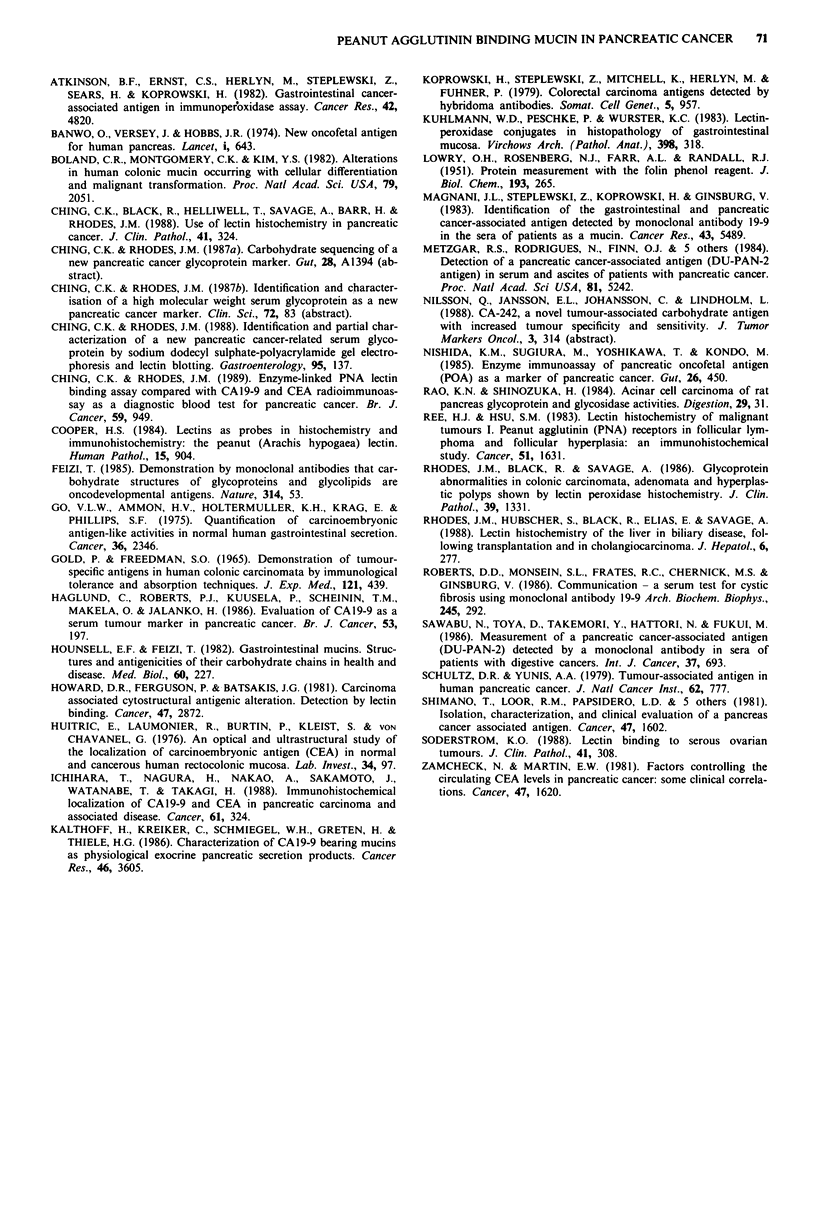# Identification of the peanut-agglutinin binding pancreatic cancer serum marker in pancreatic tissue extracts.

**DOI:** 10.1038/bjc.1990.15

**Published:** 1990-01

**Authors:** C. K. Ching, J. M. Rhodes

**Affiliations:** University Department of Medicine, Liverpool, UK.

## Abstract

**Images:**


*Br. J. Cancer* (1990), **61**, 69–71 © Macmillan Press Ltd., 1990

SHORT COMMUNICATION

### Identification of the peanut-agglutinin binding pancreatic cancer serum marker in pancreatic tissue extracts

C.K. Ching & J.M. Rhodes

*University Department of Medicine and Walton Hospital, Liverpool, UK.*

Pancreatic disease can be notoriously difficult to diagnose so there has been considerable interest recently in the development of tests for serum glycoprotein markers of pancreatic cancer. These have usually been carried out with the aid of monoclonal antibodies produced against tumour or cell line extracts. Potential serum markers for pancreatic cancer have included carcinoembryonic antigen (CEA) (Gold & Freedman, 1965; Zamcheck & Martin, 1981), pancreatic oncofetal antigen (POA) (Banwo *et al.*, 1974; Nishida *et al.*, 1985), pancreatic carcinoma associated antigen (PCAA) (Schultz & Yunis, 1979; Shimano *et al.*, 1981), DU-PAN 2 (Metzgar *et al.*, 1984; Sawabu *et al.*, 1986) and CA19-9 (Koprowski *et al.*, 1979; Magnani *et al.*, 1983; Haglund *et al.*, 1986). As most of the marker antibodies so far characterised have been found to recognise carbohydrate rather than protein epitopes (Feizi, 1985), a previous study was carried out to determine whether further tumour marker glycoproteins could be more efficiently identified using a combination of SDS-polyacrylamide gel electrophoresis and blotting with a panel of lectins chosen for their ability to identify different carbohydrate epitopes. This approach proved successful demonstrating the presence of a high molecular weight glycoprotein in approximately one-third (12/34) of the pancreatic cancer sera but in none of the 96 controls (Ching & Rhodes, 1988). Further characterisation showed this serum marker to be a mucin (Ching & Rhodes, 1987*a*). This has subsequently been developed into an enzyme-linked peanut lectin assay (PNA-ELLA) (Ching & Rhodes, 1989) for total peanut lectin binding glycoproteins in serum. This assay has proved equivalent in efficacy to CA19-9 serum radioimmunoassay and the two tests together have a combined sensitivity of 85% for pancreatic cancer (Ching & Rhodes, 1989).

Although proving useful as a serum test for pancreatic cancer, the epitope for CA19-9 is known to be present in normal pancreatic tissue (Atkinson *et al.*, 1982) and juice (Kalthoff *et al.*, 1986), bile (Albert *et al.*, 1987) and colon (Afdhal *et al.*, 1987) in a way analogous to CEA (Go *et al.*, 1975; Huitric *et al.*, 1976; Ichihara *et al.*, 1988). This study was performed to determine whether the peanut agglutinin binding glycoprotein could also be detected in normal or diseased pancreatic tissue.

Pancreatic resection specimens were obtained at laparotomy from patients with pancreatic cancer ($n = 3$, all well differentiated adenocarcinoma), normal ($n = 5$), chronic pancreatitis ($n = 4$, all due to alcohol) and one ampullary carcinoma. These were snap-frozen in liquid nitrogen and then stored at − 70°C. Polyacrylamide gradient gels (2–16%) were obtained from Pharmacia (Sweden), soybean trypsin inhibitor and peroxidase-tagged peanut agglutinin (PNA, gal 1–3 gal NAc binding) from Sigma (USA), peroxidase-tagged *Ulex europaeus* (UEA I, L-fucose binding), *Limax flavus* agglutinin (LFA, sialic acid binding) and *Griffonia simplicifolia* (GS 2, glc NAc binding) lectins from E-Y laboratory (USA) and nitrocellulose paper from Bio-Rad (UK).

Pancreatic tissue glycoprotein extraction was performed according to Rao and Shinozuka (1984) with minor modification. Approximately 1 g wet weight pancreatic tissue was used per specimen. Samples were cut into small pieces and then ultrasonicated (Ultrasonicator KS 100, Kerry Ultrasonics, UK) for 1 min in 10 ml Tris HCl (20 mM, pH 7.4)/EDTA (1 mM) buffer containing 200 μg ml$^{-1}$ soybean trypsin inhibitor. This was followed by homogenisation in a Polytron homogeniser (PCU, Kriens-Luzern, Switzerland) and then centrifugation at 13,000 *g* (Sorvall RC5, DuPont instruments, USA) for 20 min. Supernatants were discarded because preliminary analysis of concentrated supernatants from two normal pancreatic tissues did not reveal any PNA binding glycoproteins identifiable on lectin blotting from the gel. The pellets were washed × 5 and then each sample re-homogenised separately in 1 ml of the same buffer for 30 s. One ml of the homogenised tissue was mixed with 9 ml of chloroform/methanol (2:1) mixture and stirred vigorously for 30 min. The aqueous phase was separated from the lipid phase and the solid residue by centrifugation at 300 *g* (Centaur 2, Fison instrumentation services, UK) for 20 min. The aqueous phase was then concentrated by gentle evaporation under nitrogen to approximately 1/4 of its original volume and glycoprotein precipitation was carried out using nine equivalent volumes of the aqueous phase of absolute ethanol. The precipitate was obtained by centrifugation at 200 *g* for 10 min.

Glycoprotein precipitates were reconstructed in 400 μl of de-ionised, distilled water. An aliquot was used for Lowry protein estimation (Lowry *et al.*, 1951). SDS-PAGE (using approximately 100 μg protein per sample), and then high intensity transfer of proteins and glycoproteins on to nitrocellulose papers and finally identification of PNA binding glycoproteins on the blots were performed as described before (Ching & Rhodes, 1988). The high molecular weight PNA binding glycoprotein identified in the tissue extracts was then further characterised using other lectins, UEA I (25 μg ml$^{-1}$), LFA (12.5 μg ml$^{-1}$) and GS 2 (25 μg ml$^{-1}$).

The mean yield of water soluble protein obtained from one extraction step ranged between 2–4.5 mg g$^{-1}$ pancreatic tissue. A high molecular weight (approximately 3.5 million Da) PNA binding glycoprotein (lane 3, Figure 1) having identical electrophoretic mobility to the serum marker, both before and after purification, was identified in tissue extracts from 3/3 pancreatic cancers, 1/4 chronic pancreatitis and 2/5 normals. The sole ampullary carcinoma extract studied did not contain the high molecular weight glycoprotein at $3.5 \times 10^6$ Da but it showed a strong PNA binding region around $1 \times 10^6$ Da, indicating the possibility of another tumour related PNA + glycoprotein in this epithelial carcinoma. A lower molecular weight (50,000 Da approximately) peanut agglutinin binding glycoprotein was also present. This was co-purified with the 3.5 million Da glycoprotein and was still present after the 3.5 million Da glycoprotein had been cut out of the gel, eluted and rerun. Characterisation of the water soluble pancreatic tissue PNA binding 3.5 million Da

Correspondence: J.M. Rhodes, University Department of Medicine, Liverpool University, PO Box 147, Liverpool L69 3BX, UK.
Received 22 May 1989; and in revised form 4 September 1989.

**Figure 1** Result of peroxidase-PNA lectin blotting after electrophoresis on a 2–16% SDS-polyacrylamide gel showing the presence of the high molecular weight glycoprotein (arrowed) in pancreatic cancer (lane 3) and normal pancreatic (lane 5) tissue extracts having the same electrophoretic mobility as the serum marker. Lane 1, purified serum marker from pancreatic cancer; lane 2, original pancreatic cancer serum of sample loaded in lane 1; lane 4, ampullary carcinoma tissue extract. Molecular weight markers: Thy, thyroglobulin 330,000 Da; Ferl, ferritin 1 220,000 Da; Alb, albumin 67,000 Da; Cat, catalase 60,000 Da; Lac, lactate dehydrogenase 36,000 Da; Fer2, ferritin 2 18,500 Da.

glycoprotein by the use of other lectins showed that it bound LFA but not UEA I and GS 2 lectins (Table I) indicating the expression of the epitopes gal 1–3 gal NAc (blood group T antigen), and sialic acid but not L-fucose (blood group H antigen) or Glc NAc (blood group Tk antigen). Other PNA binding glycoproteins present on the blots are probably normal pancreatic epithelial structure components as we have previously shown in a lectin histochemical study that PNA binding glycoproteins other than the secreted mucus can be identified (Ching *et al.*, 1988).

This study has demonstrated that a peanut lectin (PNA) binding glycoprotein previously found in pancreatic cancer serum is also present in the pancreatic tissue itself not only in pancreatic cancer but also in benign pancreatic disease and in the normal pancreas. Both serum and tissue PNA binding glycoproteins had identical electrophoretic mobility. The lectin binding characteristics of the tissue glycoprotein extracted from benign and malignant pancreas indicate that it possesses gal 1–3 gal NAc (PNA binding) and sialic acid (LFA binding) side chains. These epitopes have been demonstrated on the serum glycoprotein (Table I) which variably bears additional epitopes namely L-fucose (H antigen, UEA I binding) and glc NAc (Tk antigen, GS2 binding) (Ching & Rhodes, 1987*b*).

The demonstration of the high molecular weight PNA binding glycoprotein in pancreatic tissue makes it very likely that the similar mucus glycoprotein previously detected (Ching & Rhodes, 1988) in pancreatic cancer sera is indeed coming from the pancreas. Shedding of mucin into serum appears to be a more regular feature of pancreatic cancer

**Table I** Lectin binding characteristics of the high molecular weight pancreatic cancer-related serum and water soluble tissue glycoproteins

| *Lectins* | *Serum glycoprotein* | *Tissue glycoprotein* |
|---|---|---|
| PNA | + | + |
| UEA I | (+) | − |
| LFA | (+) | + |
| GS2 | (+) | − |

(+), variable binding of the high mol. wt serum glycoprotein to the lectins (7/12 to UEA I and 4/12 to both LFA and GS 2) (Ching & Rhodes, 1987*b*).

compared with gastrointestinal tumours such as colonic and gastric tumours as shown by the higher rate of positive serum tests in pancreatic cancer using both enzyme-linked PNA assay and CA19-9 radioimmunoassay, even though the CA19-9 antibody was raised against a colorectal cancer cell line. In a previous study, we have shown that the PNA binding pancreatic cancer-related serum mucus glycoprotein sometimes but not always expresses the CA19-9 epitope (Ching & Rhodes, 1988). The binding sites for PNA (gal 1–3 gal NAc) and CA19-9 (sialylated *N*-fucopentaose II oligosaccharide) cannot occur on the same oligosaccharide side chains so we envisage the tumour-related mucin as a complex glycoprotein that may variably express the sialylated Lewis antigen (CA19-9 epitope) on some side chains, the PNA epitope (T antigen) on others and UEA I (H antigen) and GS 2 (Tk antigen) binding sites on yet other side chains. Simultaneous demonstration of an additional carbohydrate epitope (CA-242) on the tumour marker glycoprotein CA50 has also been reported recently (Nilsson *et al.*, 1988).

The sialylated Lewis$^a$ antigen has been found in normal colon (Afdhal *et al.*, 1987), bile (Albert *et al.*, 1987) and pancreatic juice (Kalthoff *et al.*, 1986) so seems to be a normal tissue and mucin glycoprotein that is abnormally expressed in the serum in cancer rather than an oncofetal antigen. The status of the PNA binding site (T antigen) is more uncertain. It behaves more as an oncofetal antigen in colon (Boland *et al.*, 1982; Cooper, 1984; Rhodes *et al.*, 1986), breast (Howard *et al.*, 1981), stomach (Kuhlmann *et al.*, 1983), ovary (Soderstrom, 1988) and lymphoid (Ree & Hsu, 1983) tissue. It can be predicted from the known structure of mucin that the T antigen can only be present as the base pair of the oligosaccharide side chain (Hounsell & Feizi, 1982) which is usually concealed by further glycosylation or sialylation. It seems likely that its expression at least reflects a relatively immature mucin side chain. In a previous study using lectin histochemistry, PNA binding has however been found variably in normal pancreatic cytoplasm (Ching *et al.*, 1988) and in normal large bile ducts (Rhodes *et al.*, 1988) and the study presented here confirms that it can be variably expressed in normal pancreas.

The presence of mucin in serum is perhaps surprising, but the CA19-9 epitope bearing mucin has also been found in pancreatic cancer (Haglund *et al.*, 1986) and in patients with cystic fibrosis (Roberts *et al.*, 1986). In pancreatic cancer, this might reflect either early invasion of this tumour into blood vessels or early ductal obstruction with reflux. It is clear from our study that this mucin contains at least four different oligosaccharide side chain structures and probably many more so development of a panel of monoclonal antibodies against different epitopes on this mucin may lead to the development of a more sensitive and specific test for pancreatic cancer.

C.K.C. was an Amelie Waring research fellow of the British Digestive Foundation.

### References

AFDHAL, N.H., LONG, A., TOBBIA, I., CULLEN, A. & O'DONOGHUE, D.P. (1987). Immunohistochemical CA19-9 in primary colonic polyps and polyps synchronous with colorectal cancer. *Gut*, **28**, 1049.

ALBERT, M.B., STEINBERG, W.M., HENRY, J.P., FISCHER, R.A. & GARONE, M.A. (1987). Markedly elevated levels of tumour marker CA19-9 in acute cholangitis. *Gastroenterology*, **92**, 1292 (abstract).

ATKINSON, B.F., ERNST, C.S., HERLYN, M., STEPLEWSKI, Z., SEARS, H. & KOPROWSKI, H. (1982). Gastrointestinal cancer-associated antigen in immunoperoxidase assay. *Cancer Res.*, **42**, 4820.

BANWO, O., VERSEY, J. & HOBBS, J.R. (1974). New oncofetal antigen for human pancreas. *Lancet*, **i**, 643.

BOLAND, C.R., MONTGOMERY, C.K. & KIM, Y.S. (1982). Alterations in human colonic mucin occurring with cellular differentiation and malignant transformation. *Proc. Natl Acad. Sci. USA*, **79**, 2051.

CHING, C.K., BLACK, R., HELLIWELL, T., SAVAGE, A., BARR, H. & RHODES, J.M. (1988). Use of lectin histochemistry in pancreatic cancer. *J. Clin. Pathol.*, **41**, 324.

CHING, C.K. & RHODES, J.M. (1987*a*). Carbohydrate sequencing of a new pancreatic cancer glycoprotein marker. *Gut*, **28**, A1394 (abstract).

CHING, C.K. & RHODES, J.M. (1987*b*). Identification and characterisation of a high molecular weight serum glycoprotein as a new pancreatic cancer marker. *Clin. Sci.*, **72**, 83 (abstract).

CHING, C.K. & RHODES, J.M. (1988). Identification and partial characterization of a new pancreatic cancer-related serum glycoprotein by sodium dodecyl sulphate-polyacrylamide gel electrophoresis and lectin blotting. *Gastroenterology*, **95**, 137.

CHING, C.K. & RHODES, J.M. (1989). Enzyme-linked PNA lectin binding assay compared with CA19-9 and CEA radioimmunoassay as a diagnostic blood test for pancreatic cancer. *Br. J. Cancer*, **59**, 949.

COOPER, H.S. (1984). Lectins as probes in histochemistry and immunohistochemistry: the peanut (Arachis hypogaea) lectin. *Human Pathol.*, **15**, 904.

FEIZI, T. (1985). Demonstration by monoclonal antibodies that carbohydrate structures of glycoproteins and glycolipids are oncodevelopmental antigens. *Nature*, **314**, 53.

GO, V.L.W., AMMON, H.V., HOLTERMULLER, K.H., KRAG, E. & PHILLIPS, S.F. (1975). Quantification of carcinoembryonic antigen-like activities in normal human gastrointestinal secretion. *Cancer*, **36**, 2346.

GOLD, P. & FREEDMAN, S.O. (1965). Demonstration of tumour-specific antigens in human colonic carcinomata by immunological tolerance and absorption techniques. *J. Exp. Med.*, **121**, 439.

HAGLUND, C., ROBERTS, P.J., KUUSELA, P., SCHEININ, T.M., MAKELA, O. & JALANKO, H. (1986). Evaluation of CA19-9 as a serum tumour marker in pancreatic cancer. *Br. J. Cancer*, **53**, 197.

HOUNSELL, E.F. & FEIZI, T. (1982). Gastrointestinal mucins. Structures and antigenicities of their carbohydrate chains in health and disease. *Med. Biol.*, **60**, 227.

HOWARD, D.R., FERGUSON, P. & BATSAKIS, J.G. (1981). Carcinoma associated cytostructural antigenic alteration. Detection by lectin binding. *Cancer*, **47**, 2872.

HUITRIC, E., LAUMONIER, R., BURTIN, P., KLEIST, S. & VON CHAVANEL, G. (1976). An optical and ultrastructural study of the localization of carcinoembryonic antigen (CEA) in normal and cancerous human rectocolonic mucosa. *Lab. Invest.*, **34**, 97.

ICHIHARA, T., NAGURA, H., NAKAO, A., SAKAMOTO, J., WATANABE, T. & TAKAGI, H. (1988). Immunohistochemical localization of CA19-9 and CEA in pancreatic carcinoma and associated disease. *Cancer*, **61**, 324.

KALTHOFF, H., KREIKER, C., SCHMIEGEL, W.H., GRETEN, H. & THIELE, H.G. (1986). Characterization of CA19-9 bearing mucins as physiological exocrine pancreatic secretion products. *Cancer Res.*, **46**, 3605.

KOPROWSKI, H., STEPLEWSKI, Z., MITCHELL, K., HERLYN, M. & FUHNER, P. (1979). Colorectal carcinoma antigens detected by hybridoma antibodies. *Somat. Cell Genet.*, **5**, 957.

KUHLMANN, W.D., PESCHKE, P. & WURSTER, K.C. (1983). Lectin-peroxidase conjugates in histopathology of gastrointestinal mucosa. *Virchows Arch. (Pathol. Anat.)*, **398**, 318.

LOWRY, O.H., ROSENBERG, N.J., FARR, A.L. & RANDALL, R.J. (1951). Protein measurement with the folin phenol reagent. *J. Biol. Chem.*, **193**, 265.

MAGNANI, J.L., STEPLEWSKI, Z., KOPROWSKI, H. & GINSBURG, V. (1983). Identification of the gastrointestinal and pancreatic cancer-associated antigen detected by monoclonal antibody 19-9 in the sera of patients as a mucin. *Cancer Res.*, **43**, 5489.

METZGAR, R.S., RODRIGUES, N., FINN, O.J. & 5 others (1984). Detection of a pancreatic cancer-associated antigen (DU-PAN-2 antigen) in serum and ascites of patients with pancreatic cancer. *Proc. Natl Acad. Sci USA*, **81**, 5242.

NILSSON, Q., JANSSON, E.L., JOHANSSON, C. & LINDHOLM, L. (1988). CA-242, a novel tumour-associated carbohydrate antigen with increased tumour specificity and sensitivity. *J. Tumor Markers Oncol.*, **3**, 314 (abstract).

NISHIDA, K.M., SUGIURA, M., YOSHIKAWA, T. & KONDO, M. (1985). Enzyme immunoassay of pancreatic oncofetal antigen (POA) as a marker of pancreatic cancer. *Gut*, **26**, 450.

RAO, K.N. & SHINOZUKA, H. (1984). Acinar cell carcinoma of rat pancreas glycoprotein and glycosidase activities. *Digestion*, **29**, 31.

REE, H.J. & HSU, S.M. (1983). Lectin histochemistry of malignant tumours I. Peanut agglutinin (PNA) receptors in follicular lymphoma and follicular hyperplasia: an immunohistochemical study. *Cancer*, **51**, 1631.

RHODES, J.M., BLACK, R. & SAVAGE, A. (1986). Glycoprotein abnormalities in colonic carcinomata, adenomata and hyperplastic polyps shown by lectin peroxidase histochemistry. *J. Clin. Pathol.*, **39**, 1331.

RHODES, J.M., HUBSCHER, S., BLACK, R., ELIAS, E. & SAVAGE, A. (1988). Lectin histochemistry of the liver in biliary disease, following transplantation and in cholangiocarcinoma. *J. Hepatol.*, **6**, 277.

ROBERTS, D.D., MONSEIN, S.L., FRATES, R.C., CHERNICK, M.S. & GINSBURG, V. (1986). Communication – a serum test for cystic fibrosis using monoclonal antibody 19-9 *Arch. Biochem. Biophys.*, **245**, 292.

SAWABU, N., TOYA, D., TAKEMORI, Y., HATTORI, N. & FUKUI, M. (1986). Measurement of a pancreatic cancer-associated antigen (DU-PAN-2) detected by a monoclonal antibody in sera of patients with digestive cancers. *Int. J. Cancer*, **37**, 693.

SCHULTZ, D.R. & YUNIS, A.A. (1979). Tumour-associated antigen in human pancreatic cancer. *J. Natl Cancer Inst.*, **62**, 777.

SHIMANO, T., LOOR, R.M., PAPSIDERO, L.D. & 5 others (1981). Isolation, characterization, and clinical evaluation of a pancreas cancer associated antigen. *Cancer*, **47**, 1602.

SODERSTROM, K.O. (1988). Lectin binding to serous ovarian tumours. *J. Clin. Pathol.*, **41**, 308.

ZAMCHECK, N. & MARTIN, E.W. (1981). Factors controlling the circulating CEA levels in pancreatic cancer: some clinical correlations. *Cancer*, **47**, 1620.